# Impact of transjugular intrahepatic portosystemic shunt creation on the central lymphatic system in liver cirrhosis

**DOI:** 10.1038/s41598-021-86006-7

**Published:** 2021-03-29

**Authors:** Claus Christian Pieper, Andreas Feißt, Carsten Meyer, Julian Luetkens, Michael Praktiknjo, Jonel Trebicka, Ulrike Attenberger, Christian Jansen

**Affiliations:** 1 Department of Diagnostic and Interventional Radiology, University Hospital of Bonn, Venusberg-Campus 1, 53105 Bonn, Germany; 2 Department of Internal Medicine I, University Hospital of Bonn, Venusberg-Campus 1, 53105 Bonn, Germany; 3 European Foundation for the Study of Chronic Liver Failure, Travesera de Gracia 11, 7th Floor, 08021 Barcelona, Spain; 4 Faculty of Health Sciences, University of Southern Denmark, Odense, Denmark; 5 Institute for Bioengineering of Catalonia, Barcelona, Spain; 6 Department of Internal Medicine I, University of Frankfurt, Frankfurt, Germany

**Keywords:** Liver diseases, Ascites

## Abstract

The puropse of this study was to evaluate associations of cisterna chyli (CCh) diameter with portal hemodynamics and the influence of TIPS-creation in cirrhotic patients. 93 cirrhotic patients (57 male, mean age 59 years) received CT prior to TIPS-creation. 38/93 additionally underwent post-interventional CT. CCh-diameter was measured. After categorization into patients with and without large venous collaterals (i.e. > 6 mm), data were analyzed regarding associations between CCh-diameter, clinical and portal-hemodynamic parameters and diameter-changes after TIPS-creation. Patient survival post-TIPS was analyzed. Median portosystemic pressure-gradient decreased from 20 to 9 mmHg after TIPS-creation. Large venous collaterals were observed in 59 patients. In 69/93 patients (74.2%) the CCh was detectable. Mean pre-interventional diameter was 9.4 ± 2.7 mm (large collaterals: 8.7 ± 2.0 mm, no large collaterals: 10.7 ± 3.2 mm, p = 0.003). CCh-diameter correlated strongly with pre-TIPS portal-pressure (Rs = 0.685, p = 0.0001), moderately with portosystemic-gradient (Rs = 0.524, p = 0.006), liver shear-wave-elastography (Rs = 0.597, p = 0.004) and spleen size (Rs = 0.501, p = 0.01) in patients without large collaterals, but not in patients with large collaterals. Post-TIPS CCh-diameter decreased significantly from 10.2 ± 2.8 mm to 8.3 ± 3.0 mm (p < 0.001). Patients without a detectable CCh on CT survived significantly shorter. The diameter of the CCh is associated with portal-pressure and decreases after TIPS-creation in cirrhotic patients, reflecting a portal decompression mechanism via the lymphatic system. Lack of larger central lymphatics detectable on CT may be associated with shorter survival.

## Introduction

The cisterna chyli is a saccular lymphatic structure located in the retrocrural space receiving lymphatic flow from the hepatic, intestinal and lumbar lymphatic truncs. Its cranial continuation is the thoracic duct—the main collecting lymphatic duct of the body—returning lymphatic fluid to the venous circulation via the left venous angle^1,2^.

In the liver, fluid that is filtered out of the sinusoids into the space of Disse can flow either into the interstitial space of the portal tracts, sublobular veins or the capsule^3^. From here it enters the respective lymphatic system (portal, sublobular or superficial) with primary drainage (80%) via the portal lymphatics and the hepatic trunc into the cisterna chyli. Hepatic lymph constitutes a large amount (as much as 25–50%) of lymph flow within the cisterna chyli and the thoracic duct^3^. Hepatic and intestinal lymph production can increase even further in patients with liver disease especially with portal hypertension^4–6^. Close interrelations of excessive hepatic lymph production in cirrhotic patients, portal vein pressure and ascites formation have been suggested several decades ago^7^. More recently initial imaging studies found dilated central lymphatics in patients with liver disease possibly as a consequence of increased hepatic lymph production^8–11^. However, the role of the lymphatic system and its regulatory role in liver disease are still poorly understood.

The purpose of this study was therefore to investigate associations of cisterna chyli diameter on cross-sectional imaging with clinical and portal hemodynamic parameters, the influence of portal decompression by transjugular intrahepatic portosystemic shunt (TIPS)-creation in cirrhotic patients with portal hypertension as well as patient outcome.

## Materials and methods

### Patient cohort and inclusion criteria

All consecutive patients suffering from liver cirrhosis with portal hypertension who underwent TIPS creation at our institution from 2014 to 2016 were retrospectively identified and screened. Decisions for TIPS-creation were reached in interdisciplinary consensus (internal medicine, interventional radiology).

The study was approved by the local institutional review board of the University Hospital of Bonn with a waiver for informed patient consent. All examinations were performed in accordance with the relevant guidelines and regulations. Inclusion criteria of the study were successful TIPS-creation at our institution for refractory ascites or variceal bleeding, availability of clinical and hemodynamic parameters of the patients, as well as pre-interventional contrast-enhanced CT imaging. Patients receiving liver transplantation during the post-TIPS observational period were excluded from the analysis. In a subgroup of patients additional post-interventional CT was available.

### TIPS creation

All patients underwent routine clinical and laboratory examinations prior to TIPS-creation. TIPS-creation was performed under combined ultrasound and fluoroscopic guidance with placement of a 10 mm Viatorr stent-graft (Gore Medical, Flagstaff, Arizona, USA) between the right hepatic vein and the right portal venous branch. Balloon dilatation was performed to an effective diameter of 8 to 10 mm with a non-compliant balloon (Mustang, Boston Scientific, Galway, Ireland) based on the individual needs of the patient with the goal to reduce the portosystemic gradient by half (ideally below 12 mmHg).

### Pressures measurement

Pre- and post-TIPS intravascular pressure levels (right atrial pressure, portal venous pressure, hepatic venous pressure) were measured during TIPS-creation using a 5F pigtail catheter and a pressure transducer system (Combitrans, Braun, Melsung, Germany) with a multichannel monitor (Sirecust, Siemens, Germany). Three separate measurements were performed and results are reported as the mean of the total number of valid measurements. The difference between portal and hepatic venous pressures was defined as portosystemic pressure gradient.

### Data acquisition

93 patients received pre-interventional CT before TIPS-creation and were included into the study (57 men, 36 female, mean age 59.4 [range 36–86] years) with a mean time between CT imaging and TIPS-creation of 10 ± 14 days (range 0–77). In a subgroup of 38/93 patients an additional post-TIPS CT was available (mean time between TIPS-creation and follow-up CT 118 ± 164 days (range 0–660)). Patient characteristics, indications for TIPS-creation and hemodynamic parameters were retrieved from patients’ medical records. Indications for TIPS-creation were bleeding in 33, therapy refractory ascites in 58 and a combination of both in 2 patients.

Contrast-enhanced CT scans were acquired in the portal venous phase with patients in a supine position using a 16-slice or 64-slice spiral CT scanner (Philips Medical Systems, Best, The Netherlands). Patients fasted for at least two to three hours before examination and received an enteral contrast agent (30 ml Gastrografin [Bayer Healthcare, Leverkusen, Germany] in 1 l of water) one hour before the CT.

Images were assessed with respect to (at least partial) discernibility of the cisterna chyli. The cisterna chyli was identified in the retrocrural space right of the aorta as an oblong structure isodens to water typically extending cranially into the lower caliber thoracic duct. The maximum axial diameter of the cisterna chyli was measured independently by two radiologists perpendicular to the long axis both on pre- and post-interventional CT (C.C.P, 9 years of experience, A.F, 7 years of experience; both blinded to patients’ clinical data). To this end, 3D multiplanar reconstructions of the CT data set were created offline on a clinical PACS-workstation (IMPAX, Agfa, Germany) with axial slices perpendicular to the long axis of the cisterna chyli. Measurement of the largest diameter of the cisterna chyli (outer circumference to outer circumference) was performed by hand by both readers. In patients with post-TIPS CT scans the diameter was also measured at the site of the largest diameter of the cisterna chyli. A diameter > 6 mm was defined as dilatation of the cisterna chyli^8^. Images were additionally evaluated by both radiologists in consensus according to the presence and extent of portosystemic collateral vessels (especially gastric veins, para-umbilical veins or spleno-renal shunts). In cases with visible portosystemic collaterals, the diameter of these vessels was measured perpendicular to its long axis at the largest identifiable collateral using 3D multiplanar reconstructions. A cut-off diameter of 6 mm was employed to define large versus small porto-systemic collaterals.

### Shear-wave elastography (SWE) procedure

SWE measurements of liver and spleen were performed in abdominal ultrasound with an abdominal 3.5-MHz curved-array transducer in three different regions of interest in the liver and the spleen as previously described using the Aixplorer US system (Supersonic Imagine SA; Aixplorer, Aix-en-Provence, France)^12–14^. The patients were placed in supine position with the right arm or left in maximal abduction and were requested to hold their breath for at least 5 s. A SWE measurement was considered accurate when it fulfilled the following criteria: (1) temporal stability of the selected liver area for at least three seconds before measurement, (2) two-dimensional quality confirmed by homogenous color in the region of interest and (3) a measurement region of at least 10 mm. Up to three separate measurements were performed and results reported as the mean of the total number of valid measurements.

### Statistical analysis

Statistical analysis was performed using commercially available software (IBM Corp. Released 2015. IBM SPSS Statistics for Windows, Version 23.0. Armonk, NY: IBM Corp., www.ibm.com) and GraphPad Prism (GraphPad Prism version 4.0.0 for Windows, GraphPad Software, San Diego, California USA, www.graphpad.com) was used to plot Fig. 2. Normal distribution of the data was assessed using Q-Q-plots and the Kolmogorov-Smirnov test. Statistical significance (p < 0.05) for group differences was tested with Student’s t-test or ANOVA for independent samples in case of inter-subject comparisons (large collaterals vs. no large collaterals, indications for TIPS, cause of liver disease, Child–Pugh status), and with Student's t-test for paired samples in case of intra-subject comparisons (cisterna chyli diameter pre- vs. post-TIPS). Interrelations between lymphatic vessel diameters and pre-TIPS hemodynamic parameters (portal pressure, right atrial pressure, porto-systemic gradient) were assessed using Pearson’s correlation coefficient. Inter-observer reliability of diameter measurements was investigated with the intra-class correlation coefficient (ICC). Kaplan–Meier curves and a log-rank test were used to analyze overall survival rates of patients in respect to gender, etiology of cirrhosis, bleeding vs. ascites, presence of large collaterals and detectability of the cisterna chyli). One-year overall survival rates were calculated.

## Results

### Patient characteristics

Portal-pressure was successfully lowered by TIPS-creation from a mean (± SD) of 26.5 ± 5.7 mmHg (range 11–41) to 18.7 ± 4.7 mmHg (range 8–29) and portosystemic-gradient from 19.9 ± 5.6 mmHg (range 6–34) to 8.8 ± 3.8 mmHg (range 3–24). Pre-TIPS portal pressure was slightly, but significantly higher in patients receiving TIPS for variceal bleeding than in patients treated for ascites (data not shown). None of the patients in this cohort underwent liver transplantation post-TIPS. On CT large venous collaterals (> 6 mm) were identified in 59 patients. Pre-TIPS portal pressure and portosystemic gradient did not differ significantly between patients with and without large collaterals, between different causes of cirrhosis, patients with different Child–Pugh status or patients with and without a detectable cisterna chyli (Table 1). None of the patients with post-TIPS CT showed signs of TIPS dysfunction at the time of follow-up CT. Detailed patient characteristics are summarized in Table 1.

**Table 1:** Patients Characteristics and comparison of patients with and without large venous collaterals.

Parameters	All patients n = 93	Large collaterals n = 59	No large collaterals n = 34	p
**General characteristics**				
Gender [female/male]	36/57	24/35	12/22	0.61
Age [years]	59.4 (36–85.5)	58.7 (36–84.8)	62.3 (38.4–85.5)	0.12
Etiology [viral/alcohol/other]	12/56/25	7/36/16	5/20/9	0.92
Child class [A/B/C]	14/69/10	7/45/7	7/24/3	0.50
MELD score	10 (6–24)	12 (6–24)	9.5 (6–22)	0.16
Oesophageal varices [absent/present]	15/78	8/51	7/27	0.38
Ascites [absent/mild/severe]	23/32/28	15/17/17	8/15/11	0.84
Bleeding before TIPS [yes/no]	35/58	24/35	11/23	**0.01**
HRS [yes/no ]	24/69	18/41	6/28	0.29
History of SBP [yes/no]	19/74	13/46	6/28	0.66
HE [yes/no]	21/72	19/40	2/32	**0.01**
**Laboratory values**				
Sodium [mmol/L]	138 (121–155)	139 (126–155)	137 (121–143)	0.14
Potassium [mmol/L]	4.12 (2.74–5.8)	4.06 (2.74–5.8)	4.13 (2.75–5.54)	0.65
Serum creatinine[mg/dL]	1 (0.54–8.7)	1 (0.59–2.5)	1.05 (0.54–8.7)	0.68
Blood urea nitrogen [mg/dL]	43.5 (0–168)	48 (0–168)	40 (9–148)	0.18
Total protein [g/L]	60 (16–85)	59 (16–82)	61 (20–85)	0.51
Albumin [g/L]	29 (10.6–45.7)	29 (21–45.7)	28.95 (10.6–40)	0.70
Bilirubin [mg/dL]	1.18 (0.1–4.23)	1.4 (0.1–4.23)	0.97 (0.13–4)	**0.03**
yGT [U/L]	121.5 (22–770)	113 (22–770)	128 (34–486)	0.36
ALT [U/L]	28 (10–490)	28 (13–174)	27 (10–490)	0.56
AST [U/L]	39 (11–777)	42 (11–197)	38 (14–777)	0.24
CRP [mg/L]	12.5 (0.2–120)	12.8 (0.2–114)	12.1 (0.3–120)	0.77
INR	1.2 (0.9–1.8)	1.2 (1–1.8)	1.1 (0.9–1.7)	0.17
Total white blood cell count [G/L]	7.19 (2–15.9)	7.24 (2–14)	6.97 (2.45–15.9)	0.96
Haemoglobin [g/dL]	9.6 (5.9–15.9)	9.4 (5.9–15.9)	10.35 (6.8–14.8)	0.18
Platelet count [G/L]	134 (34–697)	121 (34–332)	158.5 (45–697)	**0.03**
**Hemodynamics**				
Portal pressure [mmHg]	26 (11–46)	26 (11–46)	26 (11–39)	0.85
Central venous pressure [mmHg]	6 (0–22)	6 (0–22)	6 (1–15)	0.67
Portal pressure gradient [mmHg]	20 (6–41)	20 (8–41)	19 (6–34)	0.50
**Cisterna chyli**				
Detectabel on CT [y/n]	59/24	43/16	26/8	0.45
Diameter [mm]	9.1 (5.3–16.2)	8.7 (5.4–13.6)	10.7 (5.3–16.2)	**0.01**
**SWE**				
SWE meanliver [kPa]	41.8 (12.7–132)	40.7 (13.4–68.8)	42.9 (12.7–132)	0.81
SWE meanspleen [kPa]	37 ( 2–131)	38.2 (2–131)	35 (4–115)	0.93
Spleen size [cm]	13.5 (8–20)	14 (10–20)	12.6 (8–19)	**0.03**

Statistically significant values (p < 0.05) are shown in bold.

### Cisterna chyli diameter measurements

The cisterna chyli was detectable on pre-interventional CT images in 69/93 cases (74.2%). Patient characteristics grouped by detectability of the cisterna chyli are summarized in Table 2. The mean pre-interventional diameter of the cisterna chyli was 9.4 ± 2.7 mm (range 5.3–16.2 mm). 64/69 patients (92.8%) showed a dilated cisterna chyli (> 6 mm). The cisterna chyli was significantly larger in patients without large portosystemic collaterals compared to patients with large collaterals (10.7 ± 3.2 mm vs. 8.7 ± 2.0 mm, p = 0.003) (Table 1). There were no significant differences in cisterna chyli diameter for the whole group as well as the subgroups with and without large collaterals between different causes of cirrhosis (alcohol, hepatitis, other), indications for TIPS-creation (bleeding or ascites) or Child–Pugh status.

**Table 2:** Comparison of patients with and without a detectable cisterna chyli on CT.

Parameters	Cisterna chyli detectable on CT n = 69	Cisterna chyli not detectable on CT n = 24	p
**General characteristics**			
Gender [female/male]	20/49	16/8	0.53
Age [years]	59.4 (36–85)	65.5 (39.1–85.5)	0.12
Etiology [viral/alcohol/other]	10/43/16	2/13/9	0.35
Child class [A/B/C]	10/50/9	4/19/1	0.48
MELD score	10 (6–24)	10.5 (6–20)	0.16
Oesophageal varices [absent/present]	11/58	4/20	0.93
Ascites [absent/mild/severe]	16/25/20	7/9/8	0.56
Bleeding before TIPS [yes/no]	25/44	10/14	0.55
HRS [yes/no ]	19/50	5/19	0.79
History of SBP [yes/no]	13/56	6/18	0.54
HE [yes/no]	15/54	6/18	0.33
**Laboratory values**			
Sodium [mmol/L]	138 (121–155)	138.5 (128–148)	0.14
Potassium [mmol/L]	4.12 (2.75–5.54)	4.07 (2.74–5.8)	0.65
Serum creatinine[mg/dL]	1 (0.56–8.7)	1 (0.54–2.2)	0.68
Blood urea nitrogen [mg/dL]	45 (9–168)	37 (0–143)	0.18
Total protein [g/L]	59 (16–79)	62 (20–85)	0.51
Albumin [g/L]	28.6 (16.5–39)	31.8 (10.6–45.7)	0.7
Bilirubin [mg/dL]	1.18 (0.1–4)	1.16 (0.21–4.23)	0.32
yGT [U/L]	125.5 (26–770)	115.5 (22–585)	0.36
ALT [U/L]	28 (10–174)	29 (14–490)	0.56
AST [U/L]	38 (14–148)	45.5 (11–777)	0.24
CRP [mg/L]	11.9 (0.2–120)	14.25 (1.7–75.1)	0.77
INR	1.2 (0.9–1.8)	1.15 (1–1.5)	0.17
Total white blood cell count [G/L]	7.5 (2–15.9)	6.38 (2.07–10.4)	0.96
Haemoglobin [g/dL]	9.8 (5.9–15.9)	9.4 (6.6–14.3)	0.18
Platelet count [G/L]	121 (34–697)	144 (41–289)	**0.003**
**Hemodynamics**			
Portal pressure [mmHg]	26 (11–46)	26.5 (11–41)	0.84
Central venous pressure [mmHg]	6 (0–22)	7 (1–15)	0.66
Portal pressure gradient [mmHg]	20 (6–35)	19.5 (8–41)	0.50
**SWE**			
SWE meanliver [kPa]	41.1 (12.67–87.2)	43 (13.4–132)	0.80
SWE meanspleen [kPa]	36.3(1.36–115.4)	37.65 (4.2–131.5)	0.93
Spleen size [cm]	13.55 (8.2–20)	13 (8–16.5)	**0.03**

Statistically significant values (p < 0.05) are shown in bold.

The results of correlation analyses are summarized in Table 3. In patients with large collaterals there was no correlation between cisterna chyli diameter and portal-pressure, atrial pressure or portosystemic gradient. However, in patients without large collaterals the diameter of the cisterna chyli correlated strongly with pre-TIPS portal-pressure (Rs = 0.685, p = 0.0001), weakly with pre-TIPS atrial pressure (Rs = 0.353, p = 0.07) and moderately with the portosystemic gradient (Rs = 0.524, p = 0.006). Furthermore, cisterna chyli diameter correlated moderately with sheer wave elastography measurements of the liver (Rs = 0.597, p = 0.004) and spleen size (Rs = 0.501, p = 0.01).

**Table 3:** Correlation of cisterna chyli diameter and patients characteristics.

Parameter	All Patiens n = 93		Large collaterals n = 59		No large collaterals n = 34	
		p		P		p
**Hemodynamics**						
Portal pressure	0.225	n.s	−0.102	n.s	0.685	**0.0001**
Central venous pressure	0.134	n.s	−0.034	n.s	0.353	**0.07**
Portal pressure gradient	0.18	n.s	−0.082	n.s	0.524	**0.006**
**SWE**						
SWE mean liver	0.41	**0.002**	0.213	n.s	0.597	**0.004**
SWE mean spleen	0.355	**0.013**	0.166	n.s	0.415	**0.07**
Spleen size	0.306	**0.014**	0.261	n.s	0.501	**0.01**

Statistically significant values (p < 0.05) are shown in bold.

**Figure 1:**
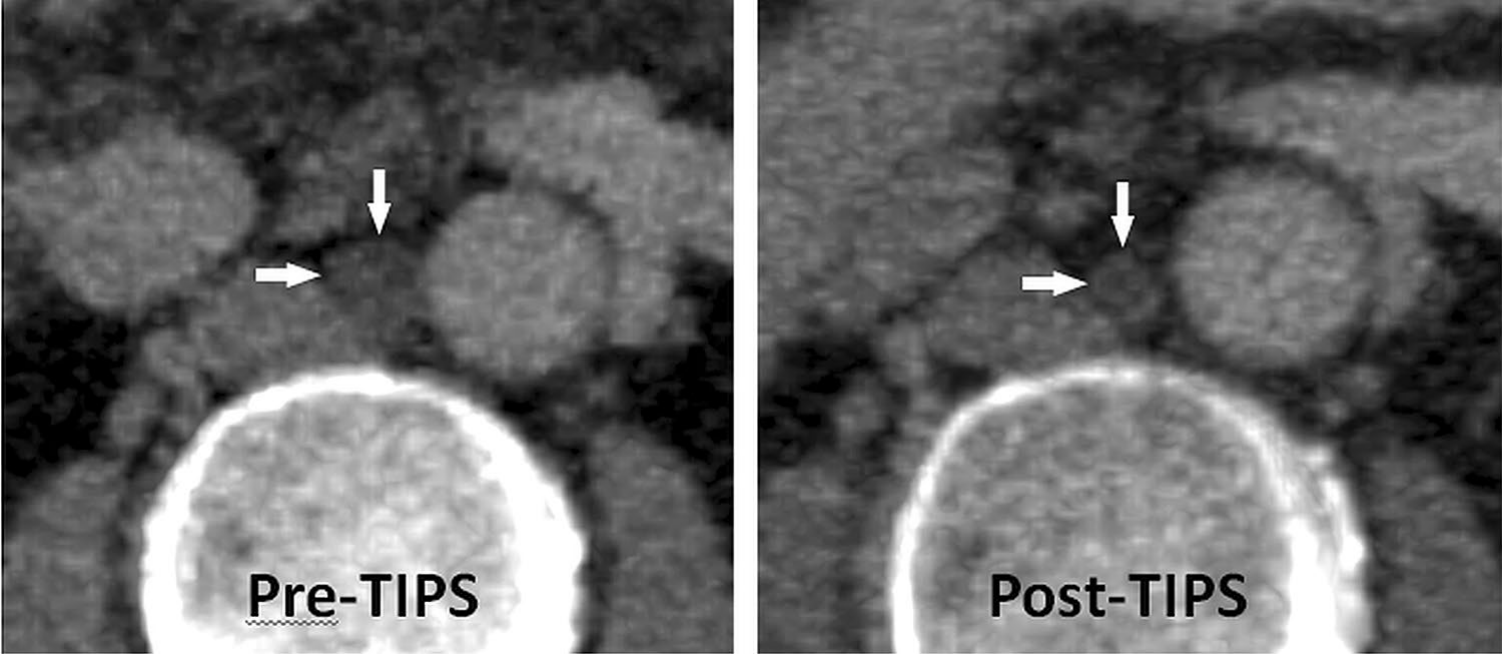
CT images of a patient before and after TIPS-creation demonstrating a considerable decrease in cisterna chyli (arrows) diameter after TIPS-creation.

**Figure 2:**
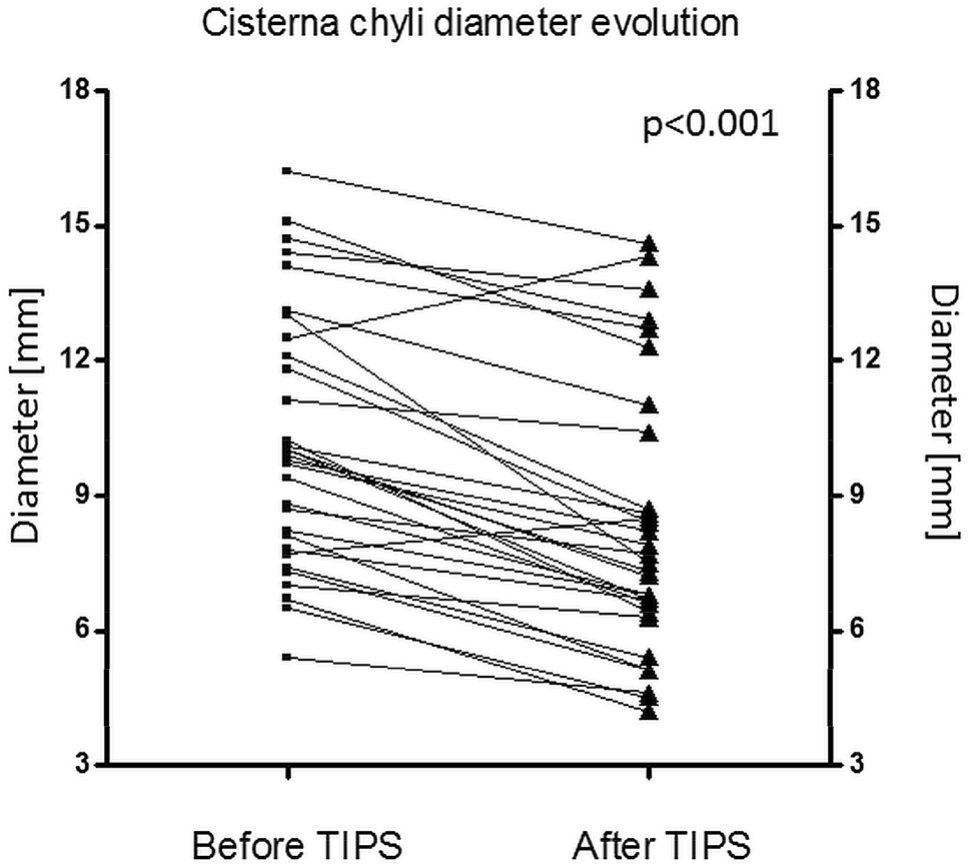
Changes in cisterna chyli diameter after TIPS creation. (GraphPad Prism version 4.0.0 for Windows, GraphPad Software, San Diego, California USA, www.graphpad.com).

On post-TIPS CT images the cisterna chyli was detectable in 31/38 cases (81.6%). In these patients with available post-TIPS follow-up CT, the diameter of the cisterna chyli decreased significantly after TIPS-creation in all but two cases from 10.2 ± 2.8 mm to 8.3 ± 3.0 mm (p < 0.001) (Figs. 1 and 2). Both patients with increasing cisterna chyli diameter showed no distinguishing clinical characteristics. One was treated for ascites, the other for recurrent bleeding. Mean diameter decrease was 1.9 ± 1.4 mm (ranging from an increase of 1.8 mm to a decrease of 5.5 mm). However, the cisterna chyli was still rated to be dilated (> 6 mm) in 26/31 cases (83.9%) after TIPS-creation.

### Interobserver reliability

Intra-class correlation coefficients indicated almost perfect agreement between the two readers with an ICC of 0.991 [0.985; 0.994] for pre-TIPS cisterna chyli diameter and 0.997 [0.994; 0.999] for post-TIPS measurements.

### Survival

**Figure 3:**
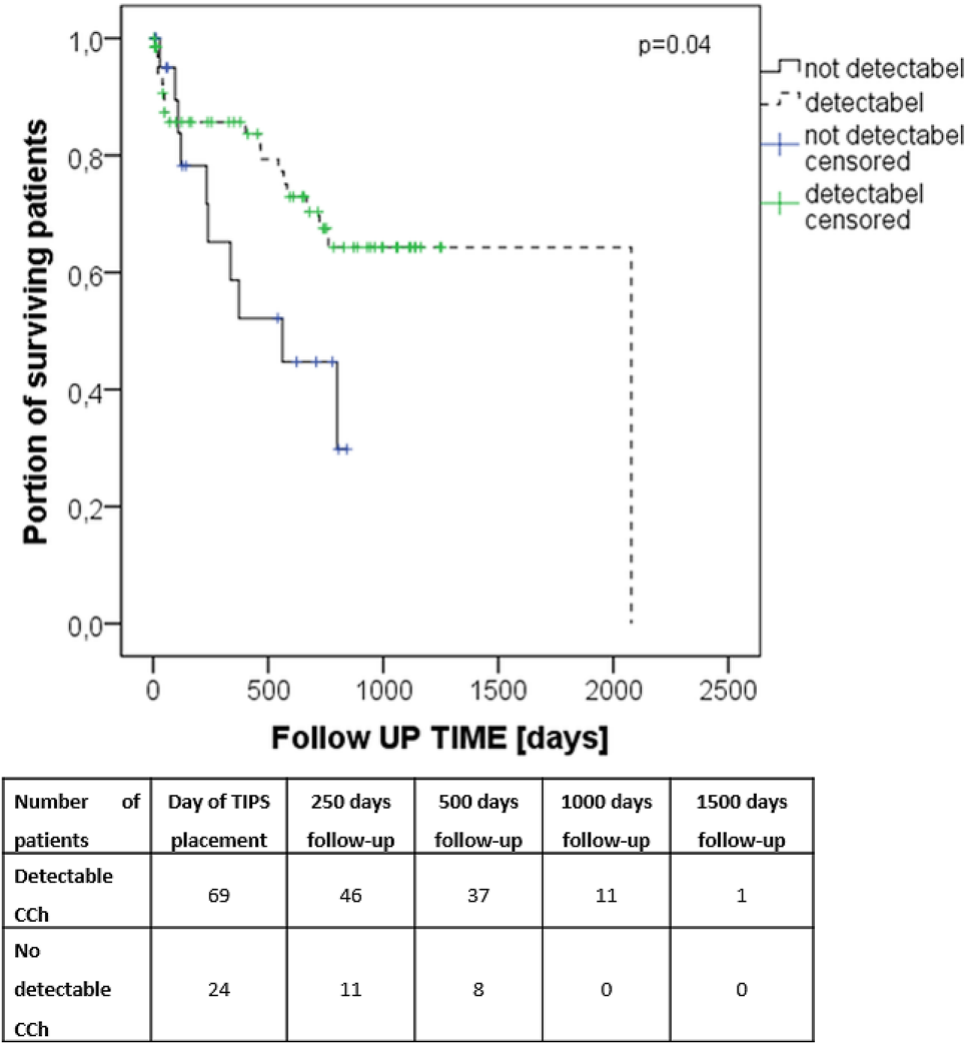
Kaplan Meier plot demonstrating overall survival in patients with detectable (dashed line) and without detectable cisterna chyli (solid line) as well as number of patients at risk at different time points. (IBM Corp. Released 2015. IBM SPSS Statistics for Windows, Version 23.0. Armonk, NY: IBM Corp., www.ibm.com).

Median overall survival after TIPS-creation was 44.8 ± 3.7 months (one-year overall survival rate 76.5%). Patients with a detectable cisterna chyli on CT survived significantly longer than those in whom the cisterna chyli was not visible on CT (49.0 ± 4.1 months vs. 17.0 ± 2.6 months; one-year overall survival rate 82.7% vs. 56.3%) (Fig. 3). There were no significant differences in survival in all other investigated grouping variables.

## Discussion

The results of the present study demonstrate that the diameter of central lymphatic vessels is closely associated with portal venous pressure in cirrhotic patients without larger venous collaterals that could provide decompression of the portal venous system. This indicates increased hepatic lymph production as a compensatory mechanism in portal hypertension. After TIPS-creation the diameter of the cisterna chyli decreases significantly, indicating decompression not only of the portal vein, but also of the lymphatic system due to decreasing hepatic lymph production.

In general, interstitial fluid and ultimately lymph is formed via filtration through the walls of blood capillaries^15,16^. Hepatic lymph primarily derives from the highly permeable sinusoids and is rich in plasma proteins^3^. In contrast to other organs, lymph production in the liver therefore is primarily dependent of hepatic blood flow and pressure levels within the sinusoids. Average portal vein pressures of 7 mmHg in healthy individuals are usually only slightly higher than average interstitial pressure within the portal tracts (5.8 mmHg)^17^, leading to a constant formation of interstitial fluid which is then primarily drained via portal lymphatics into the cisterna chyli^3^.

In healthy individuals hepatic lymph constitutes 25–50% of lymph flow within the cisterna chyli and the thoracic duct, while about 40% derives from the intestine, with variation according to the nutritional status^3^. In liver disease lymph production can increase considerably due to an increasing pressure gradient between the sinusoids and the interstitial space^4,5^. In a cirrhotic rat model hepatic lymph production increased up to 30-fold^6^.

This excessive lymph flow eventually exceeds the capacity of the central lymphatic system leading to leakage of hepatic lymph into the peritoneal cavity which in turn has been suggested as one of the factors responsible for ascites formation in cirrhotic patients^7^.

Clinically close interrelations between hepatic blood and lymphatic vessels can be deduced from several studies. Dumont and Mulholland performed surgical drainage of thoracic duct lymph in cirrhotic patients with ascites or variceal bleeding. They observed considerably elevated pressure levels within the thoracic ducts from 15–70 cm saline (11–51.5 mmHg) (normal values 6–15 cm saline [4.4–11 mmHg])^18^. Drainage of thoracic duct lymph led to a decrease in ascites volume or termination of acute variceal bleeding. However, after discontinuation of thoracic duct drainage ascites re-accumulated and patients started to bleed again.

A close connection between hepatic blood vessels and lymphatic vessels is also reflected in that fact that changes in lymphatic flow/pressure result in morphological changes of the sinusoids and vice versa, e.g. after ligation of the thoracic duct^19^. Furthermore, the area of portal lymphatics increases in patients with idiopathic portal hypertension^20^. However, as examination of microscopic lymphatic vessels within the liver in humans in vivo is not feasible, the exact association between portal vein pressure and morphological changes of lymphatic vessels remains unknown.

Various imaging techniques can nowadays be applied in vivo to examine larger central lymphatic structures like the cisterna chyli and the thoracic duct^16,21^. Several studies primarily employing oily lymphangiography have shown that both portal lymphatics and the cisterna chyli can be dilated in patients with portal hypertension^10^. Nowadays, less invasive imaging techniques like computed tomography (CT)^22^, sonography^9,11^ or magnetic resonance imaging (MRI)^10,23,24^ can reliably visualize central lymphatics in a large percentage of cases. This is in line with the results of our study with a detection rate of 74.2% for the cisterna chyli on standard contrast-enhanced CT.

Mean maximum transverse diameters of the cisterna chyli in healthy individuals differ slightly between studies, but seems to lie in the rage of 2–4 mm^10,22^. An MR-based study comparing cirrhotic patients with normal controls demonstrated that a cisterna chyli > 2 mm was identified significantly more often in patients suffering from liver cirrhosis compared to normal subjects (76% vs. 14%). Dilatation of the cisterna chyli (> 6 mm) was significantly more prevalent in patients with decompensated compared with compensated cirrhosis (54% vs. 5%), while none of the normal subjects showed a cisterna chyli > 6 mm^10^. In our study all patients suffered from decompensated liver cirrhosis. Accordingly, in 93% of patients with a visible cisterna chyli, it was dilated > 6 mm, while the remaining 7% showed a diameter between 5.3–6 mm.

When comparing patients with and without portal hypertension Ito and colleagues found that the diameter of the cisterna chyli was significantly larger with than without portal hypertension, but without observing differences in the severity of portal hypertension. This is in line with our finding that there was no correlation between cisterna chyli diameter and portal pressure when analyzing the whole patient cohort. However, pressure levels were not invasively measured in the study by Ito and colleagues, but the presence and severity of portal hypertension was defined according to the presence and diameter of portosystemic collateral vessels^8^. This definition is problematic because although large collateral vessels may indicate the presence of severe portal hypertension, blood flow in the collaterals itself can already partially decompress the portal venous system. This is corroborated by our finding that compared to patients with large collaterals, patients without large collaterals exhibited significantly larger cisterna chyli diameters showing a strong correlation with portal pressure and a moderate correlation with the portosystemic gradient (which is the more reliable measure). Accordingly, there was a moderate positive correlation of sheer wave elastography measurements and spleen size with cisterna chyli diameter. Overall these findings suggest that there is more lymph flows within the cisterna chyli in these patients as a compensatory mechanism due to the lack of decompression via larger venous collaterals.

An established treatment option for cirrhosis-related ascites is portal decompression by TIPS-creation^25–27^. After TIPS-creation cisterna chyli diameters decreased significantly in all but two patients, indicating also a decompression of the lymphatic system due to decreasing hepatic lymph production. Both patients with slightly increasing cisterna chyli diameters showed large venous collaterals before TIPS-creation but did otherwise not differ from the remaining patients.

However, the cisterna chyli was still rated to be dilated (> 6 mm) in a large number of patients after TIPS-creation. This may be seen as a compensatory mechanism even after TIPS-creation due to still elevated hepatic lymph production. If lymphatic run-off is impaired (e.g. due to strictures of the thoracic duct or elevated central venous pressure) this may give rise to recurrent ascites. This, however, was not observed in the retrospective analysis of our patient-cohort and warrants prospective investigation.

In this respect a recent case report seems to corroborate the concept of lymphatic decompression of the portal venous system^28^. They describe decompression of the thoracic duct by placing a stent-graft across the lymphovenous junction to treat acute variceal bleeding in a patient with portal hypertension. Treatment was successful without recurrence of bleeding over a period of three months after treatment.

Interestingly patients without a detectable cisterna chyli survived significantly shorter after TIPS-creation than those with a visible cisterna chyli. At present we cannot conclusively explain this finding. A possible explanation may be that the absence of large central lymphatics may lead to an impairment of compensatory lymphatic run-off in such patients and therefore to impaired overall survival. This finding certainly has to be confirmed in larger patient cohorts as the cisterna may also not have been detectable on some CT-scans due to technical reasons (insufficient contrast between the adjacent anatomical structures). However, the cisterna chyli is known to be anatomically absent in 20–30% of patients^29^ which is in agreement of our detection rate. Further prospective studies with complete morphological and functional imaging work-up of the central lymphatic system may therefore be interesting in patients with therapy-refractory or recurrent ascites after TIPS-creation.

The results of our study are limited by its retrospective character with inherent methodological problems. First, results of measurements could not be compared to a gold standard as patients included into the study did not receive examinations specially dedicated to the depiction of the lymphatic system (such as conventional or MR lymphangiography). However, the successful use of contrast enhanced CT to visualize the cisterna chyli has already been described in literature and measurement results in our study showed almost perfect agreement between the two independent radiologist. Second, times between pre-TIPS CT and TIPS-creation, as well as between TIPS-creation and follow-up CT were not standardized. Although the mean time between pre-TIPS CT and TIPS-creation was only 10 days, it was as long as 3 months in single patients, so that the correlation of measured diameter (CT) and pressure (TIPS-creation) may be limited. Especially time intervals between intervention and follow-up CT differed so that pressure levels at the time of follow-up CT were not available as none of the patients had an indication for secondary invasive TIPS catheterization. We therefore refrained from correlation analysis of post-interventional pressure levels and cisterna chyli diameter. Furthermore, we cannot exclude a selection bias in the group of patients receiving post-TIPS follow-up CT as this cohort may represent especially the subgroup of patients with a stable clinical course after TIPS. More homogenous time intervals between CT and TIPS-creation would be needed to overcome this limitation. However, patients are not routinely submitted to CT scans before and after TIPS-creation due to radiation protection issues. Third, lymph flow within the cisterna chyli and the thoracic duct is known to increase after ingestion of food. The exact nutritional status of the patients at the time of CT could not be evaluated retrospectively, however, all CT scans showed largely empty stomachs except for enteral contrast agent. Fourth, diameter measurements may be influenced by random contraction waves of the cisterna chyli which have been observed in up to 11% of patients in a previous MR based study^8^. To exclude effects of these contraction waves repeated examinations would have been necessary, but cannot be performed in clinical routine. Width of the cisterna chyli is further affected by patient position with significantly larger cisterns in patients in a standing or sitting compared to a supine position^30^. However, all patients in our study were examined in the same supine position. Finally, the detection rate of the cisterna chyli on CT is certainly limited by available image quality. “Not detectable” therefore is not equivalent to “aplastic”, but rather a combination of an actual aplasia of the cistern as well as lymph vessels that are too small to image on CT.

Due to the exploratory nature of this initial retrospective study without dedicated lymphatic imaging, we did not perform a multivariate survival analysis. Further prospective studies with lymphatic imaging are warranted to further evaluate the impact of the lymphatic system on patient survival after TIPS-creation.

## Conclusion

In conclusion the cisterna chyli can be confidently identified in about 75% of patients with end stage liver cirrhosis and portal hypertension on standard contrast enhanced CT. The diameter of the cisterna chyli was dilated in the majority of patients and was closely associated with portal pressure levels in patients without large venous collaterals that could have partly decompressed the portal venous system. Cisterna chyli diameter decreases significantly after TIPS-creation indicating decreasing hepatic lymph production and decompression of the lymphatic system after TIPS-creation. Further investigation into the association between lack of a detectable cisterna chyli and shorter overall survival after TIPS-creation is warranted.
